# Functional Characterization of Sex Pheromone Neurons and Receptors in the Armyworm, *Mythimna separata* (Walker)

**DOI:** 10.3389/fnana.2021.673420

**Published:** 2021-04-28

**Authors:** Chan Wang, Bing Wang, Guirong Wang

**Affiliations:** ^1^State Key Laboratory for Biology of Plant Diseases and Insect Pests, Institute of Plant Protection, Chinese Academy of Agricultural Sciences, Beijing, China; ^2^Guangdong Laboratory of Lingnan Modern Agriculture, Shenzhen, Genome Analysis Laboratory of the Ministry of Agriculture, Agricultural Genomics Institute at Shenzhen, Chinese Academy of Agricultural Sciences, Shenzhen, China

**Keywords:** *Mythimna separata*, pheromone receptors, odorant receptor neurons, single sensillum recording, *Xenopus* oocyte

## Abstract

Pheromone receptors (PRs) of moths are expressed on the dendritic membrane of odorant receptor neurons (ORNs) housed in the long trichoid sensilla (TS) of antennae and are essential to sex pheromone reception. The function of peripheral neurons of *Mythimna separata* in recognizing sex pheromones is still unclear. In this study, electroantennogram recordings were performed from male and female antennae of *M. separata*, and showed that the major component of sex pheromones, (*Z*)-11-hexadecenal (Z11–16:Ald), evoked the strongest response of male antennae with significant differences between sexes. Single sensillum recording was used to record responses of neurons housed in TS of male *M. separata*. The results revealed four types of TS with three neurons housed in each type, based on profiles of responses to sex pheromone components and pheromone analogs. ORN-B of type-I TS was specifically tuned to the major sex pheromone component Z11–16:Ald; ORN-Bs in type-III and type-IV TSs were, respectively, activated by minor components (*Z*)-11-hexadecen-1-yl acetate (Z11–16:OAc) and hexadecenal (16:Ald); and ORNs in type-II TS were mainly activated by the sex pheromone analogs. We further cloned full-length sequences of six putative *PR* genes and an *Orco* gene. Functional characterization of PRs in the *Xenopus* oocyte system demonstrated that male antennae-biased MsepPR1 responded strongly to (*Z*)-9-tetradecenal (Z9-14:Ald), suggesting that *MsepPR1* may be expressed in type-II TS. MsepPR6 was exclusively tuned to (*Z*)-9-tetradecen-1-yl acetate (Z9–14:OAc). MsepPR2 and MsepPR4 showed no responses to any tested components. Female antennae-biased MespPR5 was broadly tuned to Z9–14:Ald, Z9–14:OAc, Z11–16:Ald, and (*Z*)-11-hexadecen-1-ol (Z11–16:OH). Our results further enriched the sex pheromone recognition mechanism in the peripheral nervous system of moth *M. separata*.

## Introduction

Pheromone-based sexual communication in moths has become an excellent model system for investigating the molecular mechanism of sensory perception because of the surprisingly high specificity in insect olfaction (Gould et al., 2010; Leal, 2013; Liu et al., 2013). Peripheral reception of pheromones in moths involves multiple proteins in male antennae, including pheromone binding proteins (PBPs), pheromone receptors (PRs), pheromone degrading enzymes, and sensory neuron membrane proteins (SNMPs; Leal, 2005; Rutzler and Zwiebel, 2005; Touhara and Vosshall, 2009).

In Lepidoptera, females release pheromone molecules that are specific and attractive to conspecific males at incredibly low concentrations over long distances (Ando et al., 2004). The species-specificity of pheromone production and recognition limits hetero-specific mating behaviors (Linn and Roelofs, 1995). Moth sex pheromones are generally a blend of pheromone components detected by odorant receptor neurons (ORNs) housed in trichoid sensilla (TS) of male antennae (Kaissling, 1986; Heinbockel and Kaissling, 1996; Hallem et al., 2004; Hansson and Stensmyr, 2011). In general, PRs expressed on the dendrite membrane of ORNs in the peripheral olfactory system of male antennae play a significant role in detecting conspecific sex pheromones (Vogt, 2005; Tanaka et al., 2009).

In early studies, two PRs of *Bombyx mori*, BmorOR1, and BmorOR3, were deorphanized (Sakurai et al., 2004; Nakagawa et al., 2005). Later, many PRs from moth species were characterized by homologous cloning technology, including *Heliothis virescens*, *Manduca sexta*, *Helicoverpa armigera*, *Spodoptera exigua*, *Sesamia inferens*, *Spodoptera litura*, *Helicoverpa assulta*, *Grapholita molesta*, *Operophtera brumata*, *Cydia pomonella*, *Ostrinia furnacalis*, *Lampronia capitella*, *Athetis lepigone*, and *Spodoptera frugiperda* (Zhang and Löfstedt, 2015). A phylogenetic analysis showed that PR clades were highly conserved and divided into different groups in Lepidoptera species (Zhang et al., 2017). In a recent study, a novel lineage of PRs clade that was part of a distinct early diverging lineage for detecting sex pheromones was characterized in *Spodoptera littoralis* and *Dendrolimus punctatus*, providing new insights into sex communication in moths (Bastin-Héline et al., 2019; Shen et al., 2020). These receptors have a potentially critical function in maintaining the integrity of species as well as in adaptation and evolution.

The oriental armyworm, *Mythimna separata* (Lepidoptera: Noctuidae) is a serious pest in many parts of the world. It distributed widely in eastern Asia and Australia, and there have been recent outbreaks in north China (Jiang et al., 2014a). The gluttonous and omnivorous characteristics of *M. separata* larvae cause huge damage to cereal crops annually, including maize, cotton, wheat, and corn (Jiang et al., 2014b). In addition, it is a migratory pest that can migrate about 1,000 km per season (Liu et al., 2017). In general, sex pheromones can be used as an efficient and environmentally friendly way of studying behavioral regulation and monitoring populations in pest control (Witzgall et al., 2010; Jiang et al., 2011). Different geographical populations of *M. separata* have different compositions and proportions in the sex pheromone gland (Takahashi et al., 1979; Zhu et al., 1987; Kou et al., 1992; Wang and Liu, 1997; Lebedeva et al., 2000; Fónagy et al., 2011; Song et al., 2017). The sex pheromone component of female *M. separata* in north China is a blend of (*Z*)-11-hexadecen-1-ol (Z11–16:Ald), (*Z*)-11-hexadecen-1-ol (Z11–16:OH), (*Z*)-11-hexadecenyl acetate (Z11–16:OAc), and hexadecenal (16:Ald; Jiang et al., 2019). Field trapping experiments showed that Z11–16:Ald alone resulted in high male moth captures (Wei and Pan, 1985; Zhu et al., 1987; Jung et al., 2013; Chen et al., 2018). A subsequent wind tunnel experiment indicated that single component Z11–16:Ald could sufficiently induce male sexual behaviors and elicit electrophysiological activity of male antennae in the gas chromatography-electroantennographic detection analyses (Jiang et al., 2019). This result revealed that Z11–16:Ald was the major component while the other three were minor components of sex pheromones in geographical populations of *M. separata* in north China.

Sex pheromone components are usually detected in TS of male moth (Kaissling, 2004). The ultrastructure of antennal sensilla of *M. separata* has been studied by scanning electron microscopy (SEM). And three dendrites were observed in the TS by the transmission electron microscopy (TEM), indicated three neurons housed in TS (Chang X. Q. et al., 2015). Recently, several studies have identified multiple chemosensory genes in the *M. separata* antennal transcriptome (Chang et al., 2017; He et al., 2017; Du et al., 2018; Jiang et al., 2019). In the geographic population of Kyoto, two PRs were deorphanized and one of them, MsOR1, was mainly tuned to sex pheromone component Z11–16:OAc (Mitsuno et al., 2008). In the geographic population of northern China, a previous analysis of antennae transcriptome identified *Orco* and six putative *PR* genes according to tissue expression and phylogenetic relationships. These genes were named *MsepPRs*, of which *MsepPR1*, *MsepPR3*, and *MsepPR4* appear to be specifically expressed in male antennae, while *MsepPR5* was highly expressed in female antennae (Du et al., 2018). Recent work published by Jiang et al. (2019) indicated that the major sex pheromone component Z11–16:Ald activated MsepOR3 and the cumulus of the macroglomerular complex (MGC) in the central nervous system, also studied olfactory coding of sex pheromones in the males *M. separata*. However, the function of peripheral neurons in discriminating minor sex pheromone components and pheromone analogs is still unclear.

In this study, we selected four sex pheromone components Z11–16:Ald, Z11–16:OAc, Z11–16:OH, and 16:Ald, consistent with the sex pheromone blend of *M. separata* identified by Jiang et al. (2019) and four pheromone analogs Z9–14:OAc, Z9–16:Ald, Z9–14:Ald, and Z9–14:OH, to focus on the sex pheromone recognition mechanism in the peripheral neuron system of *M. separata*. Firstly, we measured electroantennography (EAG) responses of male and female *M. separata* antennae to sex pheromone components. Secondly, we recorded multiple ORNs housed in TS of male moth using single sensillum recording (SSR). Different types of TS were sorted according to functional profiles. Thirdly, we cloned six full-length *PR* genes and an *Orco* gene identified from published antennae transcriptomes using rapid amplification of cDNA ends (RACE) technology. Finally, we identified the functions of MsepPRs using the *Xenopus* oocyte heterologous expression system and two-electrode voltage clamp. Our results enriched the mechanism of sex pheromone recognition in the peripheral nervous system of *M. separata*.

## Materials and Methods

### Insects

Mythimna *separata* adults were caught in the field in Xinxiang, Henan Province, China (35°18′ N, 113°55′ E). The larvae were reared on an artificial diet at a temperature of 25 ± 1°C, humidity of 75 ± 10% and photoperiod of 14:10 h (light:dark) in the Institute of Plant Protection, Chinese Academy of Agricultural Sciences. Pupae were distinguished based on sex and placed in separate cages before eclosion. The adults were fed with fresh 10% glucose water.

### Chemical Compounds

Four sex pheromone components Z11–16:Ald (CAS:53939-28-9), Z11–16:OH (CAS:56683-54-6), Z11–16:OAc (CAS:34010-21-4), 16:Ald (CAS:629-80-1), and four pheromone analogs (*Z*)-9-hexadecenal (Z9-16:Ald; CAS:56219-04-6), Z9–14:Ald (CAS:53939-27-8), (*Z*)-9-tetradecen-1-ol (Z9-14:OH; CAS:35153-15-2), Z9–14:OAc (CAS:16725-53-4) used in this study were purchased from Nimrod Inc. (Changzhou, China; purity ≥ 96%).

### Electroantennogram Recordings

The electrophysiological recordings of whole male and female antennae in response to four sex pheromone components and pheromone analogs were performed according to the standard technique (Cao et al., 2016). The components used in the EAG assay were dissolved in paraffin oil and diluted to 10 μg/μL. A piece of filter paper (0.5 × 5 cm) loaded with 10 μL pheromones was used as a stimulus, and paraffin oil was used as a control. 3-day-old moths were tested and signals from antennae were amplified with a 10 × AC/DC headstage preamplifier (Syntech, Kirchzarten, Germany) and further acquired with an Intelligent Data Acquisition Controller (IDAC-4-USB; Syntech, Kirchzarten, Germany). Signals were recorded with Syntech EAG-software (EAGPro 2.0). After subtracting the responses of the control, data were analyzed using the Student’s *t*-test (El-Sayed et al., 2009).

### Single Sensillum Recordings

Trichoid sensilla of antennae of 3-day-old male adults were used for recordings. TS in the basal, middle, and proximal parts of the antennae were recorded for each antenna. Individuals were restrained in a remodeled 1 mL plastic pipette tip with an exposed head fixed by dental wax, and the antenna from one side was attached to a coverslip with double-sided tape. A tungsten wire was inserted into one compound eye of the moth as a reference electrode, and a recording electrode was inserted into the base of each TS after sharpening the tip with 40% KNO_2_ solution. The recording electrode was attached to an olfactory probe (Syntech) under a Leica Z16 APO microscope at 920 × magnification, and action potentials were amplified by a 10 × AC/DC preamplifier (Syntech).

The sex pheromone components were dissolved in paraffin oil at a concentration of 100 μg/μL and were stored at −20°C. The working concentrations were prepared by a serial dilution from 200 to 0.01 μg/μL (200, 100, 10, 1, 0.1, 0.01 μg/μL). Paraffin oil was used as a negative control. The chemicals were dripped on a filter paper strip (0.5 × 5 cm) inserted into a pasteur pipette (15 cm long). Purified and humidified air flow set at 1.2 L/min continuously blew toward the antenna through a 14 cm-long metal tube controller (Syntech, Kirchzarten, Germany). The fixed antennae were exposed to a 300 ms stimulus air pulse controlled by a Syntech stimulus controller (CS-55, Syntech, Kirchzarten, Germany). AC signals were recorded for 10 s using a data acquisition controller (IDAC-4, Syntech, Kirchzarten, Germany). Action potentials were digitized and displayed on a computer screen using Autospike software (Syntech). Responses were calculated as the difference of spike-number between the 1 s before the stimulus delivery point and 1 s after (Chang et al., 2016).

### RNA Extraction and cDNA Synthesis

Total RNAs were extracted using Trizol reagent (Invitrogen, Carlsbad, CA, United States) following the manufacturer’s instructions from male’ and female’ antennae. The quantity and quality of RNA were, respectively, detected using a Nanodrop ND-1000 spectrophotometer (NanoDrop Products, Wilmington, DE, United States) and gel electrophoresis. Single first strand cDNAs were synthesized using RevertAid First Strand cDNA Synthesis Kit (Thermo Scientific, United States).

### Phylogenetic Analysis and Cloning of Pheromone Receptors

The sequences of *MsepOrco* and six *MsepPR* genes were identified by antennal transcriptomic analysis in our previous study (Du et al., 2018). For the phylogeny, we aligned six MsepPRs with previously identified PRs in *M. separata* (Mitsuno et al., 2008; Jiang et al., 2019) and four other closely related species in Lepidoptera, including *H. virescens* (Wang et al., 2010), *H. armigera* (Liu et al., 2013), *H. assulta* (Jiang et al., 2014c), and *S. litura* (Zhang and Löfstedt, 2015). Sequences were aligned using DNAMAN 7.0 (Lynnon Bioisoft, United States). Phylogenetic and molecular evolutionary analyses were conducted using MEGA 6.0 (Tamura et al., 2013).

To get the full-length open reading frame sequences of the candidate *MsepPRs*, 3’ and 5’ RACE were performed using a SMARTer RACE cDNA Amplification kit (Clontech, Mountain View, CA, United States). Specific primers were designed using Primer Premier 5.0 software (PREMIER Biosoft International, CA, United States) and were listed in Supplementary Table 1. The polymerase chain reactions were carried out under the following conditions: 95°C for 3 min; 35 cycles of 98°C for 10 s, 55°C for 30 s, 72°C for 1.5 min; 72°C for 10 min. PCR products were run on a 1.0% agarose gel, and sequences were verified by DNA sequencing after ligation into the cloning vector pEASY-Blunt (TransGen Biotech, China).

### PR Expression in *Xenopus* Oocytes and Two-Electrode Voltage-Clamp Recordings

Open reading frames of *PR* genes were subcloned into the pT_7_Ts vector based on the specific restriction enzyme digestion sites (Supplementary Table 1). Plasmids were fully linearized with corresponding restriction enzymes. cRNAs were synthesized using mMESSAGE mMACHINE SP6 kit (Thermo Scientific, United States). Purified cRNAs were diluted at a concentration of 2 μg/μL and stored at −80°C. Mature healthy oocytes were treated with 2 mg/mL collagenase type I in washing buffer for 1-2 h at room temperature (Liu et al., 2013). A mixture of 27.6 ng of *MsepPR* cRNA and *MsepOrco* cRNA was microinjected into oocytes. After 3–5 days of incubation at 18°C in 1 × Ringer’s buffer (96 mM NaCl, 2 mM KCl, 5 mM MgCl_2_, 0.8 mM GaCl_2_, and 5 mM HEPES; pH 7.6 adjusted by NaOH) supplemented with 5% dialyzed horse serum, 50 μg/mL tetracycline, 100 μg/mL streptomycin, and 550 μg/mL sodium pyruvate, the injected oocytes were recorded with a two-electrode voltage clamp.

Four sex pheromone components and four pheromone analogs were dissolved in dimethyl sulfoxide (DMSO) to form the stock solutions (1 M) and stored at −20°C. Stock solutions were diluted in 1 × Ringer’s buffer up to work concentration of 10^–4^ M. The negative control was 1 × Ringer’s buffer. Currents induced by sex pheromone components were recorded using an OC-725C oocyte clamp (Warner Instruments, Hamden, CT, United States) at a holding potential of −80 mV. Between each stimulus, the oocytes were washed with 1 × Ringer’s buffer to return to a stable baseline (Wang et al., 2010). Data were acquired and analyzed with Digidata 1440A and pCLAMP 10.0 software (Axon Instruments Inc., Union City, CA, United States).

### Statistics and Data Analysis

Statistics were mainly analyzed using SPSS 22.0 (IBM Inc., Chicago, IL, United States) and bar-graphs were created with GraphPad Prism 5 (GraphPad Software, Inc., CA, United States). Data of EAG responses were analyzed using Student’s *t*-test (*P* < 0.05 or *P* < 0.01). Odor responses were normalized using linear model for each neuron and clustered using the agglomerative hierarchical clustering method with HemI 1.0 (Deng et al., 2014). Data of two-electrode voltage-clamp recordings were analyzed using one-way ANOVA followed by LSD test (*P* < 0.05). Dose-response data were analyzed using GraphPad Prism 5 (GraphPad Software, Inc., CA, United States). Amino acid sequence alignment was performed using DNAMAN 7.0 (Lynnon Bioisoft, United States) and the phylogenetic tree was constructed using MEGA 6.0 (Tamura et al., 2013) and visualized and modified using FigTree 1.4.4 (Institute of Evolutionary Biology, University of Edinburgh, United Kingdom).

## Results

### Electroantennogram Responses

**FIGURE 1:**
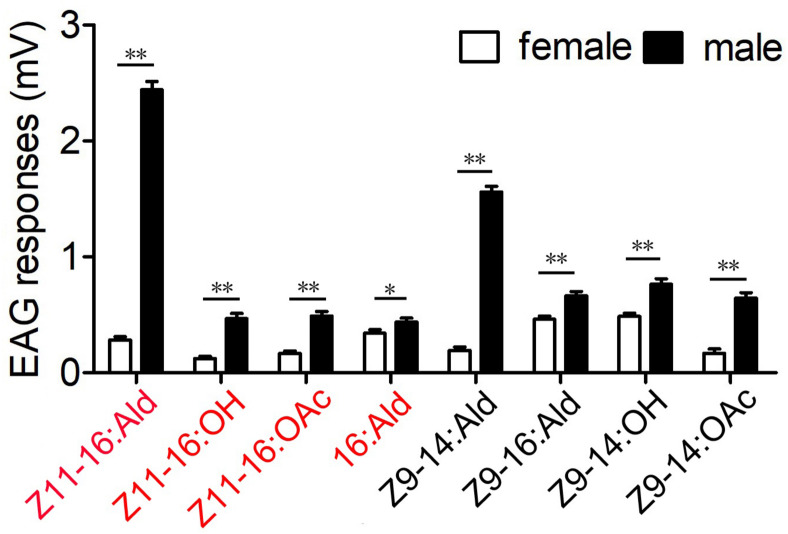
Electroantennography (EAG) responses from antennae of male and female *M. separata* to four sex pheromone components (red) and four pheromone analogs (black). The EAG response values marked with asterisks represent significant differences between sexes (*, **, respectively, indicate significant differences under 0.05 and 0.01 levels, determined by a Student’s *t*-test). Error bars indicate SEM (*n* = 13).

In this study, four sex pheromone components and four pheromone analogs were chosen to evaluate the antennal EAG responses of male and female *M. separata*. The results showed that all tested compounds elicited EAG responses of male antennae at the dose of 100 μg (Figure 1). Major sex pheromone component Z11–16:Ald evoked the strongest EAG responses from antennae of male moths and showed highly significant differences between sexes (*P* < 0.01). The responses of male antennae were also significantly evoked by analog Z9–14:Ald. Minor pheromone components Z11–16:OH, Z11–16:OAc, 16:Ald and other analogs induced weak EAG responses. The EAG responses showed significant differences between male and female antennae for all tested compounds (*P < 0.05 or **P < 0.01; Figure 1).

### Responses of ORNs to Sex Pheromones

**FIGURE 2:**
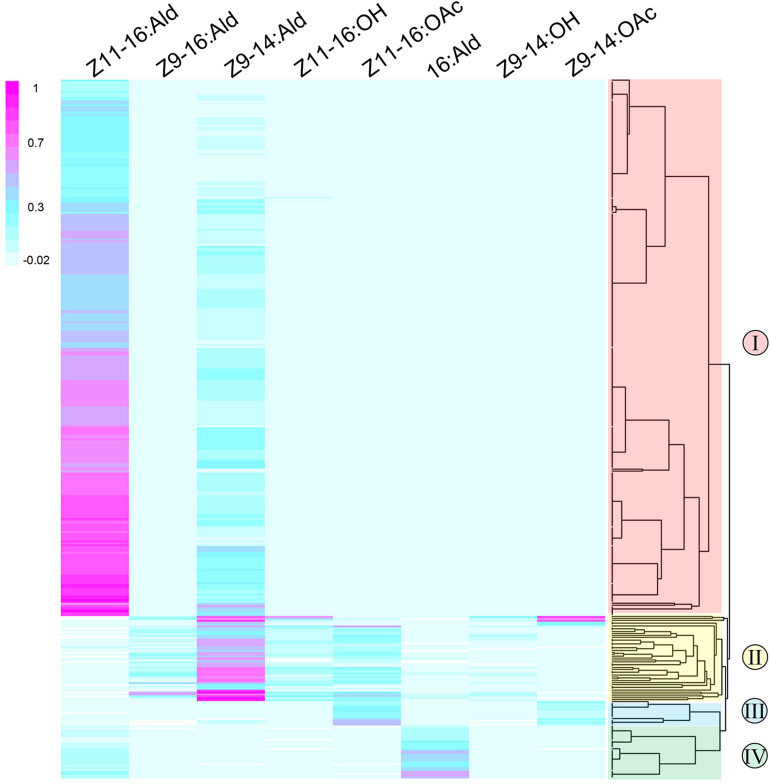
Response profiles of functional ORNs housed in 403 TS in the antennae of male *M. separata* (*y*-axis) in response to four sex pheromone components and four pheromone analogs (*x*-axis). The classification dendrogram was generated using the agglomerative hierarchical clustering method, leading to four functional TS types (I, II, III, and IV). Responses are normalized using linear model and color coded for each neuron. Magenta indicates a strong response of ORNs to odorants; blue, weak excitation; light blue, no response; white indicates that spontaneous spiking activity was reduced compared to baseline.

**FIGURE 3:**
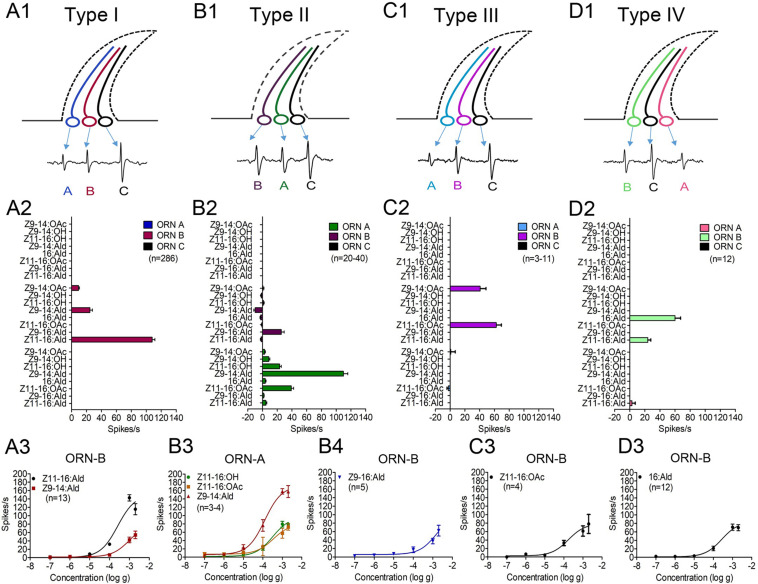
Response profiles of three distinct ORNs (-A, -B, -C) and dose-response curves of functional ORNs housed in four types of TS (type I, II, III, and IV) on the antennae of male *M. separata* in response to four sex pheromone components and four pheromone analogues. **(A1,B1,C1,D1)** Distinct ORNs housed in four types of TS. **(A2,B2,C2,D2)** Response profiles of distinct ORNs housed in four types of TS. The amount of each stimulus was 1 mg. The responses of ORN-B of type-I TS were 101 ± 2 spikes/s (*n* = 286) for Z11–16:Ald and 27 ± 1 spikes/s (*n* = 286) for Z9–14:Ald. The ORN-A of type-II TS was activated by Z9–14:Ald, Z11–16:OAc, Z11–16:OH, with responses of 110 ± 6 spikes/s (*n* = 40), 39 ± 3 spikes/s (*n* = 40), and 23 ± 2 spikes/s (*n* = 40), respectively. Responses of ORN-B were 26 ± 4 spikes/s (*n* = 40) for Z9–16:Ald. The ORN-B of type-III TS was activated by Z11–16:OAc and Z9–14:OAc with responses of 63 ± 7 spikes/s (*n* = 11) and 41 ± 8 spikes/s (*n* = 3), respectively. The ORN-B of type-IV TS was activated by 16:Ald and Z11–16:Ald with responses of 60 ± 7 spikes/s (*n* = 12) and 24 ± 4 spikes/s (*n* = 12). **(A3,B3,B4,C3,D3)** Dose-response curves of functional ORNs. The order of the neurons among sensilla types and color code were random. Error bars indicate mean ± SEM.

Single sensillum recordings were extensively performed on the TS of male moths to test neuronal responses to four sex pheromone components and four pheromone analogs at the dose of 1 mg. In total, ORNs housed in 466 TS at different positions from all segments of male antennae were recorded. The functional patterns were clustered into four distinct types (named I, II, III, and IV) of TS (Figure 2), and each of them housed three neurons, named A, B and C based on the amplitude size of the spike (Figures 3-A1, -B1, -C1, -D1). Neuron A had the smallest action potential, followed by neuron B, while neuron C had the largest amplitude. We found that neurons housed in 403 TS were activated (Figure 2), and a great majority of those were type-I (*n* = 293), followed by type-II (*n* = 70), and type-IV (*n* = 27). Much less abundant responses were recorded with type-III (*n* = 13).

The activities of ORNs housed in different sensilla types revealed peripheral coding of sex pheromone components of male *M. separata*. ORN-B of type-I TS exhibited highly specific responses to the major component Z11–16:Ald and slight responses to analog Z9–14:Ald, while no responses of ORN-A and -C were activated to tested pheromone components (Figure 3-A2 and Supplementary Figure 1-A1). We next measured dose-response curves of neurons housed in type-I TS to their active compounds across a dose range from 10^–7^ *g* to 2 × 10^–3^ *g*, showing that ORN-B in type-I TS are more sensitive to the major component Z11-16:Ald with an EC_50_ value of 2.58 × 10^–4^ *g* and low sensitivity to analog Z9-14:Ald with an EC_50_ value of 1.04 × 10^–3^ *g* (Figure 3-A3 and Supplementary Figures 1-A2, -A3).

Type-II TS were divided into two sub-groups based on responses of ORN-B to analog Z9–14:OAc. In sub-group 1, there was no response of ORN activated by Z9–14:OAc, showing that ORN-A and -B of type-II TS mainly responded to minor pheromone components and their analogs. ORN-A in type-II TS were strongly activated by analog Z9–14:Ald, followed by minor components Z11–16:OAc and Z11–16:OH. ORN-B were activated by analog Z9–16:Ald (Figure 3-B2 and Supplementary Figure 1-B1). The dose-response curves of ORN-A and -B are, respectively, shown in Figures 3-B3,-B4. ORN-A was more sensitive to analog Z9–14:Ald (EC_50_ = 1.25 × 10^–4^ *g*) than minor components Z11–16:OH (EC_50_ = 3.23 × 10^–4^ *g*) and Z11–16:OAc (EC_50_ = 3.45 × 10^–4^ *g*; Supplementary Figures 1-B2, -B3, -B4, 3-B3), while ORN-B was less sensitive to analog Z9–16:Ald with an EC_50_ value of 1.55 × 10^–3^ *g* (Supplementary Figures 1-B5, 3-B4). The sub-group 2 (in a few cases) showed that ORN-A was activated by Z9–14:Ald, Z11–16:OH, Z9–14:OAc and Z9–14:OH (Figure 2 and Supplementary Figure 2); ORN-B was also activated by analog Z9–16:Ald (Supplementary Figures 2-A, -B). Dose-response curves of ORN-A showed sensitivity to analog Z9–14:OAc with an EC_50_ value of 1.31 × 10^–3^ *g* (Supplementary Figures 2-C, -D).

ORN-B of type-III TS responded to minor component Z11–16:OAc and analog Z9–14:OAc (Figure 3-C2 and Supplementary Figure 1-C1). ORN-B of type-IV TS mainly responded to minor component 16:Ald and had a small response to the major component Z11–16:Ald (Figure 3-D2 and Supplementary Figure 1-D1). In type-III and -IV TS, there were neuronal responses to several concentrations of minor components Z11–16:OAc (EC_50_ = 1.71 × 10^–4^ *g*; Figure 3-C3 and Supplementary Figure 1-C2) and 16:Ald (EC_50_ = 2.76 × 10^–4^ *g*; Figure 3-D3 and Supplementary Figure 1-D2), respectively, suggesting increasing firing rate in a dose-related manner.

### Gene Cloning and Sequence Analysis of *M. separata* PRs

**FIGURE 4:**
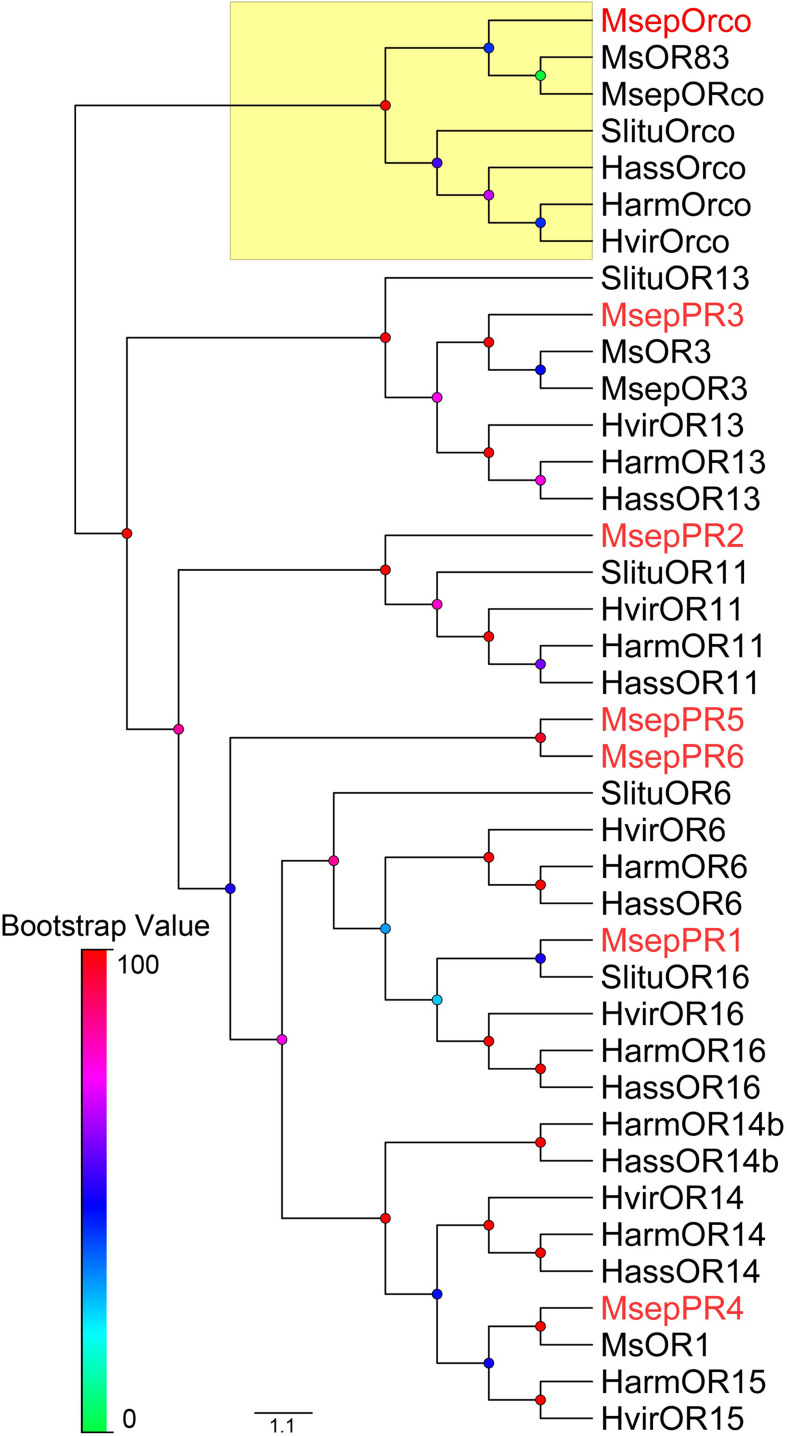
Phylogenetic tree of PRs from *M. separata* and four other Lepidoptera species. Msep: *M. separata* (red), Msep: *M. separata* (black, Jiang et al., 2019, 2020), Ms: *M. separata* (black, Mitsuno et al., 2008), Harm: *Helicoverpa armigera*, Hass: *H. assulta*, Hvir: *Heliothis virescens*, and Slitu: *Spodoptera litura.* The Orco clade is marked with yellow. This tree was inferred using the neighbor-joining method. Node support was assessed with 1,000 bootstrap replicates.

We cloned the full-length of *MsepOrco* and six *MsepPR* genes (*MsepPR1*, *MsepPR2*, *MsepPR3*, *MsepPR4*, *MsepPR5*, *MsepPR6*) from published *M. separata* antennal transcriptome, which separately encode 473, 432, 435, 424, 445, 431, and 434 amino acids (Du et al., 2018). The amino acid sequences of MsepOrco and six MsepPRs from this study were used to construct a phylogenetic tree with two previously identified PRs from *M. separata* of the Kyoto geographic population (Mitsuno et al., 2008), seven PRs from *H. armigera*, six PRs from *H. assulta*, four PRs from *S. litura* and six PRs from *H. virescens*, and their Orco sequences, clearly showing a highly conserved Orco group and another PR clade (Figure 4). The PRs in this study clustered in different clades as follows: MsepPR1 and OR16; MsepPR2 and OR11; MsepPR3 and OR13; MsepPR4 and OR15; in addition to MsepPR5 and MsepPR6 (Figure 4). The identities of amino acid sequences in OR11, OR13, OR15, and OR16 clades were quite different (Supplementary Figure 3). The OR11 sequences from five closely related species were conserved with 80.59–81.96% similarity (Supplementary Figure 3-B), while other clades were relatively divergent (Supplementary Figures 3-A, -C, -D). We also compared the identities of amino acid sequence of MsepPR3 with two geographic populations in north China (MespOR3, Jiang et al., 2019), which showed 99.76% similarity with only one amino acid varying (Supplementary Figure 4). The amino acid sequences of MsepOrco between the two geographic populations were exactly the same (Supplementary Figure 4). All of the *PR* genes identified from *M. separata* in different geographic populations are listed in Supplementary Table 2.

### Functional Characterization of *M. separata* PRs in the *Xenopus* Oocyte Expression System

**FIGURE 5:**
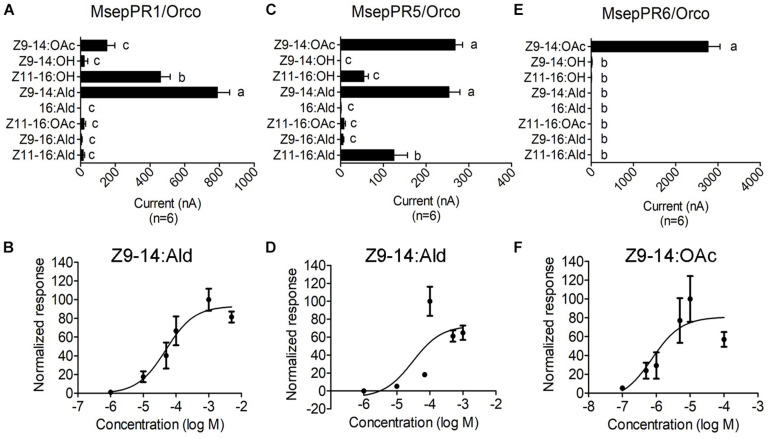
Response profiles of MsepPR1/Orco, MsepPR5/Orco, and MsepPR6/Orco to four sex pheromone components and four pheromone analogs in *Xenopus* oocyte system. **(A,C,E)** Response profiles of MsepPR1/Orco, MsepPR5/Orco, and MsepPR6/Orco in response to 10^–4^ M solution of stimuli. Error bars indicate mean ± SEM (*n* = 6). Comparisons between groups were made using ANOVA followed by LSD’s test. Different letters above the error bars indicated significant difference at the 0.05 level. **(B)** Dose-response curves of MsepPR1/Orco expressed in *Xenopus* oocyte to Z9–14:Ald. EC_50_ = 4.90 × 10^–5^ M. Error bars indicate mean ± SEM (*n* = 10). **(D)** Dose-response curves of MsepPR5/Orco to Z9–14:Ald. EC_50_ = 3.04 × 10^–5^ M. Error bars indicate mean ± SEM (*n* = 5). **(F)** Dose-response curves of MsepPR6/Orco to Z9–14:OAc. EC_50_ = 7.46 × 10^–7^ M. Error bars indicate mean ± SEM (*n* = 8). Responses are normalized by defining the average response as 100.

In this study, responses of five PRs to sex pheromone components were recorded using a two-electrode voltage clamp. In total, four sex pheromone components and four pheromone analogs at the concentration of 10^–4^ M were tested. The responses of MsepPR1 with a high expression level in male antenna were mainly tuned to analog Z9–14:Ald (787 ± 71 nA), followed by minor sex pheromone component Z11–16:OH and analog Z9–14:OAc with current values of 460 ± 56 and 151 ± 47 nA, respectively, (Figure 5A and Supplementary Figure 5-A1). In a dose-response experiment, MsepPR1/MsepOrco was sensitive to Z9–14:Ald at concentrations as low as 10^–6^ M with an EC_50_ value of 4.90 × 10^–5^ M (Figure 5B and Supplementary Figure 5-A2). MsepPR5 had a high expression level in female antennae and was tuned to analogs Z9–14:Ald (254 ± 26 nA) and Z9–14:OAc (268 ± 18 nA), and was also slightly activated by the major sex pheromone component Z11–16:Ald and minor component Z11–16:OH with the current values of 125 ± 32 and 54 ± 11 nA, respectively, (Figure 5C and Supplementary Figure 5-B1). Dose-response study showed MsepPR5/MsepOrco was sensitive to Z9–14:Ald at concentrations as low as 10^–6^ M with an EC_50_ value of 3.04 × 10^–5^ M (Figure 5D and Supplementary Figure 5-B2). MsepPR6 expressed in male antenna was specifically tuned to analog Z9–14:OAc with a large current value of 2764 ± 285 nA (Figure 5E and Supplementary Figure 5-C1). Dose-response study showed MsepPR6/MsepOrco was sensitive to Z9–14:OAc at concentrations as low as 10^–7^ M with an EC_50_ value of 7.46 × 10^–7^ M (Figure 5F and Supplementary Figure 5-C2). MsepPR2 and MsepPR4 showed no response to any tested compounds (Supplementary Figure 6).

## Discussion

Courtship and mating behaviors in moths largely rely on sex pheromones released from females, which are artificially applied to lure males and for population monitoring in pest control. Male moths could recognize intra- and inter-specific sex pheromones to multiply and keep species isolation. In this study, four functional types of TS were characterized. Type-I TS was responsible for the major pheromone component Z11–16:Ald, type-II TS was responsible for the minor pheromone component Z11–16:OH, behavioral antagonist Z9–14:Ald and some inter-specific pheromones. Type-III and -IV TS recognized minor pheromone components Z11–16:OAc and 16:Ald, respectively. Subsequently, putative PRs were functionally characterized. Our results help to improve the olfactory coding of sex pheromones and inter-specific pheromones in the peripheral neuron system.

**FIGURE 6:**
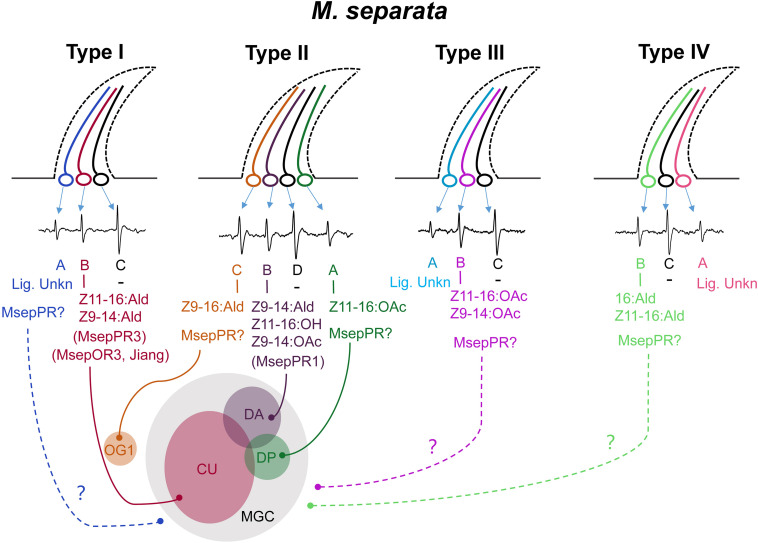
A schematic diagram of olfactory coding of sex pheromones and analogs in male *M. separata* at the peripheral and central nervous system. Four functional types of TS were characterized. The classification of neurons housed in type-II TS is modified by our SSR recording and the reported results by Jiang et al. (2020). Neuron of type-II TS activated by Z11–16:OAc is divided as a active one. In total, six ORNs are separately activated by major and minor sex pheromone components and their analogs. “–” indicates the largest spontaneous action potential without ligands identified. “Lig. Unkn” indicates no ligand has been characterized for a specific neuron by SSR recording. The curves with solid lines indicate candidate project pathways. The curves with dotted lines indicate unknown project pathways. MGC, macroglomerular complex; CU, cumulus; DA, dorso-anterior; DP, dorso-posterior; and OG1, ordinary glomerulus 1.

Functions of ORNs housed in each type of TS were characterized using the SSR technique. Unlike the results of two types of TS identified by Jiang et al. (2020), we characterized four functional types of TS housed 12 ORNs in *M. separata*, implying that peripheral coding in olfaction of *M. separata* was more complicated than in other Lepidoptera moths such as *H. armigera* and closely related species (Wu et al., 2013; Zhang et al., 2013; Sakurai et al., 2014; Chang et al., 2016; Liu et al., 2018). Our results show that five ORNs are separately activated by major and minor sex pheromone components and their analogs (Figure 3). Of those, ORN-B housed in type-I TS is considered the neuron type detecting the major pheromone component and represents the most frequently occuring neuron type in our recordings. This result is consistent with a recent study by Jiang et al. (2020). Otherwise, their previous work indicated that MsepOR3 (equal to MsepPR3 in this study, see Supplementary Figure 4) was tuned to the major component Z11–16:Ald and analog Z9–14:Ald from *M. separata*, inferring that an *MsepOR3*-expressing neuron may be ORN-B of type-I TS based on identical function and the axons from this neuron projects to the cumulus (CU) of the MGC in male antennal lobes (ALs; Figure 6). Moreover, since a majority of ORN-B of type-I TS are recorded in response to the major component Z11–16:Ald, it might explain why the largest EAG activities are observed from male antennae (Figure 1).

In our study, the response profiles of neurons of type-II TS are quite similar with those in B type sensilla reported by Jiang et al. (2020). However, the number of active neurons between these two studies is different. The results by Jiang et al. (2020) showed that three neurons and three different subunits of MGC were activated by Z9–16:Ald, Z11–16:OAc, Z9–14:Ald and Z11–16:OH, respectively, according to the evidence provided by SSR technology and *in vivo* optical imaging methods, while our study had its limitation for characterizations of active neurons at peripheral neuron system based on the amplitude size of the spike. We preliminarily identified two active neurons, one (ORN-A) was activated by Z9–14:Ald, Z11–16:OH and Z11–16:OAc, another (ORN-B) was activated by Z9–16:Ald. In fact, neurons activated by Z11–16:OAc and Z11–16:OH in our study were hard to distinguish according to the shape and size of the spike. Therefore, we integrated the results in our studies and in reported studies by Jiang et al. (2019, 2020), and divided neurons of type-II TS into four ORNs. We drew a schematic diagram in Figure 6, showing that Z11–16:OAc activated ORN-A, Z9–14:Ald and Z11–16:OH activated ORN-B, Z9–16:Ald activated ORN-C of type-II TS, respectively.

In *H. armigera*, Z9–14:Ald is an agonist at low concentrations and becomes an antagonist at high concentrations when in combination with other compounds (Gothilf et al., 1978; Kehat and Dunkelblum, 1990; Zhang et al., 2012; Wu et al., 2015). Further functional characterization showed that Z9–14:Ald was recognized by HarmOR14b, HarmOR16, and HarmOR6 expressed in Type B or Type C TS (Liu et al., 2013; Chang et al., 2016; Wang et al., 2018). In this study, we found that analog Z9–14:Ald could strongly elicit the ORN-B of type-II TS of *M. separata*, and also ORN-B of type-I TS with mild sensitivity (Figure 6), corresponding to a recent study that Z9–14:Ald activated the CU and the dorso-anterior (DA) of the MGC in ALs of male *M. separata* (Jiang et al., 2020). Wind tunnel assay further indicated that addition of Z9–14:Ald at the ratio of 1:1, 1:10, and 1:100 greatly reduced the attractiveness of *M. separata* to Z11–16:Ald (Jiang et al., 2020), suggesting that Z9–14:Ald plays vital roles in species isolation of *M. separata* and its function as antagonist within noctuid moths is conserved in the evolution. We also found that MsepPR1 was homologous with OR16 from *H. virescens*, *H. armigera*, *H. assulta*, and *S. litura*, and shared 70.02–73.61% identities of conserved amino acid sequences (Wang et al., 2011; Liu et al., 2013; Jiang et al., 2014c; Zhang and Löfstedt, 2015), indicating that they may have the same function. The *Xenopus* oocyte *in vitro* study showed that MsepPR1 was a PR for detecting Z9–14:Ald, Z11–16:OH and Z9–14:OAc, which was consistent with the SSR recording from the ORN-B of type-II TS, especially in sub-group 2, suggesting that MsepPR1 may be expressed in this neuron with its axon projecting to the DA of MGC (Figure 6).

In our experiments, MsepPR6-expressing oocytes responded highly to analog Z9–14:OAc, also known as the interspecific pheromones of *S. frugiperda* (Groot et al., 2008), *Agrotis segetum* (Zhang and Löfstedt, 2013), *S. exigua* (Liu et al., 2013), *S. litura* (Zhang and Löfstedt, 2015), and *A. lepigone* (Zhang et al., 2019). Thus, MsepPR6 may be involved in reproductive isolation of *M. separata*. However, we did not find potential neurons elicited by Z9–14:OAc alone. In addition, the expression level of *MsepPR6* gene was low in male antennae (Du et al., 2018). We therefore speculate that the *MsepPR6-*expressing neuron is not characterized in our experiments. However, identifying more functions needs support from experiments such as *in situ* hybridization and CRISPR-Cas9 technology.

In a previous study, Z11–16:OAc and Z11–16:OH were isolated at a ratio of 8:1 from female abdominal tips of *Leucania separata* Walker, a geographic population of Japan (Takahashi et al., 1979). Later, MsOR1 was identified and characterized as a major PR responding to Z11–16:OAc (Mitsuno et al., 2008). The phylogenetic analysis in this study revealed that MsOR1 (geographic population of Kyoto) and MsepPR4 (geographic population of north China) were homologous to HvirOR15 and HarmOR15 in the PR15 clade, which had no ligands based on previous studies (Wang et al., 2011; Liu et al., 2013). In our experiments, MsepPR4 did not respond to any sex pheromone components or their analogs but shared 91.24% identity of amino acid sequences with MsOR1. The functional differentiation of PRs in different geographic populations could be explained by variation of key sites of amino acid sequences. Besides, responses in ORN-A of type-II TS, ORN-B of type-III TS and ORN-B of type-IV TS were elicited by the minor sex pheromones Z11–16:OAc and 16:Ald, respectively. However, the PRs expressed in these types of TS and subunits of the MGC in ALs are still unknown (Figure 6). This phenomenon may be explained by limiting conditions such as the lack of other co-factor PBPs and SNMPs in the *Xenopus* oocyte system or unidentified PRs in a novel lineage of the PR clade (Große-Wilde et al., 2006; Benton et al., 2007; Sun et al., 2013; Chang H. et al., 2015; Wang et al., 2016; Bastin-Héline et al., 2019; Shen et al., 2020).

In this study, we found that the number of ORNs, which had the largest spontaneous action potential recorded by a tungsten wire electrode (ORN-C of type-I, -III, -IV TS, and ORN-D of type-II TS, see Figure 6), housed in each type of TS is quite low. Similar action potentials were also recorded in coeloconic sensilla of *M. separata* (Tang et al., 2020), *Manduca sexta* (Zhang et al., 2019), and *Drosophila melanogaster* (Benton et al., 2009). We speculate that non-responding neurons in TS to express for example IRs and to respond to other odor classed than pheromones. Further study is needed to confirm it.

It is worth mentioning that female moths could also detect sex pheromones emitted from inter- and intra-specific females. Several behavioral assays revealed that female moths detected such sex pheromones to repel conspecific females, reduce mating, increase movements and flight activity to a significant degree, and improve chances of progeny survival (Ellis et al., 1980; Saad and Scott, 1981; Stelinski et al., 2014; Holdcraft et al., 2016). In this study, we tested the function of MsepPR5, which was specifically highly expressed in female antennae (Du et al., 2018), showing that it could be activated by the major sex pheromone component Z11–16:Ald, antagonist Z9–14:Ald and interspecific pheromone component Z9–14:OAc, espeacially from the genus *Spodoptera* (Tamaki et al., 1973; Teal et al., 1985; Fumiaki et al., 1993). MsepPR5 is hypothesized to play an important role in sex pheromone detection of female moths, and to be involved in repellent behavior through perception of high population density in order to reduce resource competition among progeny (Pearson et al., 2004). However, the molecular mechanism of olfactory recognition of sex pheromones in female *M. separata*, is still unknown and requires follow-up experiments.

## Data Availability Statement

The datasets presented in this study can be found in online repositories. The names of the repository/repositories and accession number(s) can be found in the article/Supplementary Material.

## Author Contributions

CW, BW, and GW designed the experiments. CW performed the experiments and analyzed the data. BW and GW contributed reagents and materials. CW, BW, and GW wrote and revised the manuscript. All authors contributed to the article and approved the submitted version.

## Conflict of Interest

The authors declare that the research was conducted in the absence of any commercial or financial relationships that could be construed as a potential conflict of interest. The handling editor declared a past co-authorship with one of the authors, GW.
