# Supplementary material for: Functional Characterization of Sex Pheromone Neurons and Receptors in the Armyworm, Mythimna separata (Walker)
Source: Front Neuroanat. 2021 Apr 28;15:673420. doi: 10.3389/fnana.2021.673420 (PMC8113758; doi:10.3389/fnana.2021.673420)
Supplement: Supplementary file 1 [file Table_1.pdf]

Table S1. Primers for gene clones

Experiments	Primer name	Primer sequences (5'-3')
RACE	PR1-5GSP	GGCCCCAAAGATGAAAAACCATTAGTGA
	PR1-5NGSP	ATAGCCCCTCATTTGAGTCACCGTATCG
	PR1-3GSP	CTATGCTGCAGGACGATACAAAAGTGGG
	PR1-3NGSP	TCACTAATGGTTTTTCATCTTTGGGGCC
	PR6-5GSP	CGGTGTGTCTCCATTGCGTAATCAGAGT
	PR6-5NGSP	CGTGAAAGCAACAAATCCCATAAGCGTA
	PR6-3GSP	TACGCTTATGGGATTTGTTGCTTTCACG
	PR6-3NGSP	ACTCTGATTACGCAATGGAGACACACCG
Cloning of full-length genes	MsepOrco-F	ATGATGACCAAAGTGAAGGC
	MsepOrco-R	TTACTTGAGTTGCACCAACAC
	MsepPR1-F	ATGACTCTAAGATCATTCCTGTTC
	MsepPR1-R	TCACACGCTGCGTAGAAAA
	MsepPR2-F	ATGCTTGCTCGACTCGAAA
	MsepPR2-R	TTAGCTGTCGAACGTGCG
	MsepPR4-F	ATGTCTTATAATTCGCGCAGT
	MsepPR4-R	TTACATGCTTTTAAGAAAAGTGAA
	MsepPR5-F	ATGGAAAGAATTAAAAAAATACTCC
	MsepPR5-R	TTACATGCTGCGAAGGACT
	MsepPR6-F	ATGCAAAGATTGAAAAAAAAGTT
	MsepPR6-R	CTACTTGCTTCGAAGGAAAGTA
Construction of expression vector	MsepOrco-T7F	TCA<u>GGGCCC</u>**GCCACC**ATGATGACCAAAGTGAAGGC
	MsepOrco-T7R	TCA<u>GCGGCCGC</u>TTACTTGAGTTGCACCAACAC
	MsepPR1-T7F	TCA<u>GGGCCC</u>**GCCACC**ATGACTCTAAGATCATTCCTGTTC
	MsepPR1-T7R	TCA<u>GCGGCCGC</u>TCACACGCTGCGTAGAAAA
	MsepPR2-T7F	TCA<u>GGGCCC</u>**GCCACC**ATGCTTGCTCGACTCGAAA
	MsepPR2-T7R	TCA<u>GCGGCCGC</u>TTAGCTGTCGAACGTGCG
	MsepPR4-T7F	TCA<u>GGGCCC</u>**GCCACC**ATGTCTTATAATTCGCGCAGT
	MsepPR4-T7R	TCA<u>GCGGCCGC</u>TTACATGCTTTTAAGAAAAGTGAA
	MsepPR5-T7F	TCA<u>GGGCCC</u>**GCCACC**ATGGAAAGAATTAAAAAAATACTCC
	MsepPR5-T7R	TCA<u>GCGGCCGC</u>TTACATGCTGCGAAGGACT
	MsepPR6-T7F	TCA<u>GGGCCC</u>**GCCACC**ATGCAAAGATTGAAAAAAAAGTT

MsepPR6-T7R	TCAGCGGCCGCCTACTTGCTTCGAAGGAAAGTA

Note: "F" indicates forward strand; "R" indicates reverse strand; "TCA" indicates protective base, the underlined bases indicate restriction recognition sites, the bolded bases indicate Kozak sequence.

Table S2. *PR* genes identified from *M. separata* in different geographic populations.

Geographic population	*PR* genes identified from *M. separata*							References
Kyoto	*MsOR83*	-	-	*MsOR3*	*MsOR1*	-	-	Mitsuno et al., 2008
Xinxiang	*MsepOrco*	*MsepPR1*	*MsepPR2*	*MsepPR3*	*MsepPR4*	*MsepPR5*	*MsepPR6*	Du et al., 2018
Baoding	*MsepORco*	*MsepOR2*	*MsepOR4*	*MsepOR3*	*MsepOR1*	*MsepOR5*	*MsepOR6*	Jiang et al., 2019

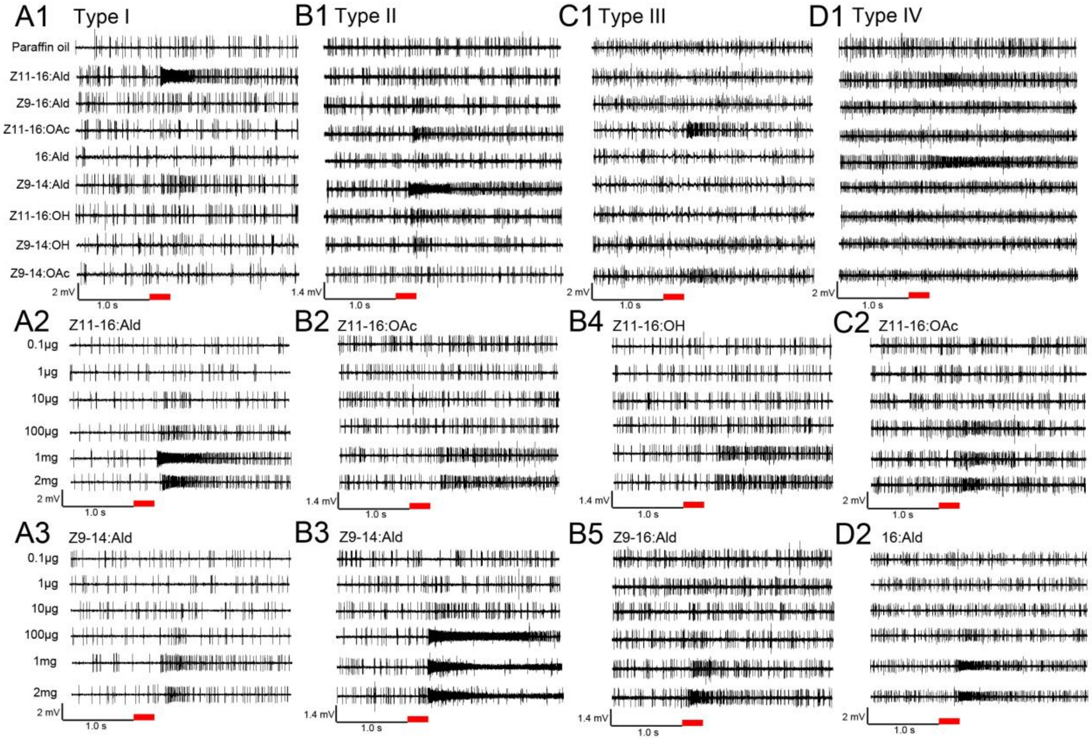

Figure S1. SSR traces of four types of TS (type I, II, III and IV) on the antennae of male *M. separata* in response to four sex pheromone components and four pheromone analogues. (A1, B1, C1, and D1). Traces showing that ORN-B of type-I TS were activated by Z11-16:Ald and Z9-14:Ald. ORN-A of type-II TS were activated by Z11-16:OH, Z11-16:OAc and Z9-14:Ald. ORN-B of type-II TS were activated by Z9-16:Ald. ORN-B of type-III TS were activated by Z11-16:OAc and Z9-14:OAc.ORN-B of type-IV TS were activated by 16:Ald and Z11-16:Ald. The amount of each stimulus was 1 mg. Traces showing ORNs were activated across a range of doses from 0.1 μg to 2 mg (A2, A3, B2, B3, B4, B5, C2, and D2). The red bold line represents 0.3 s odour stimulation.

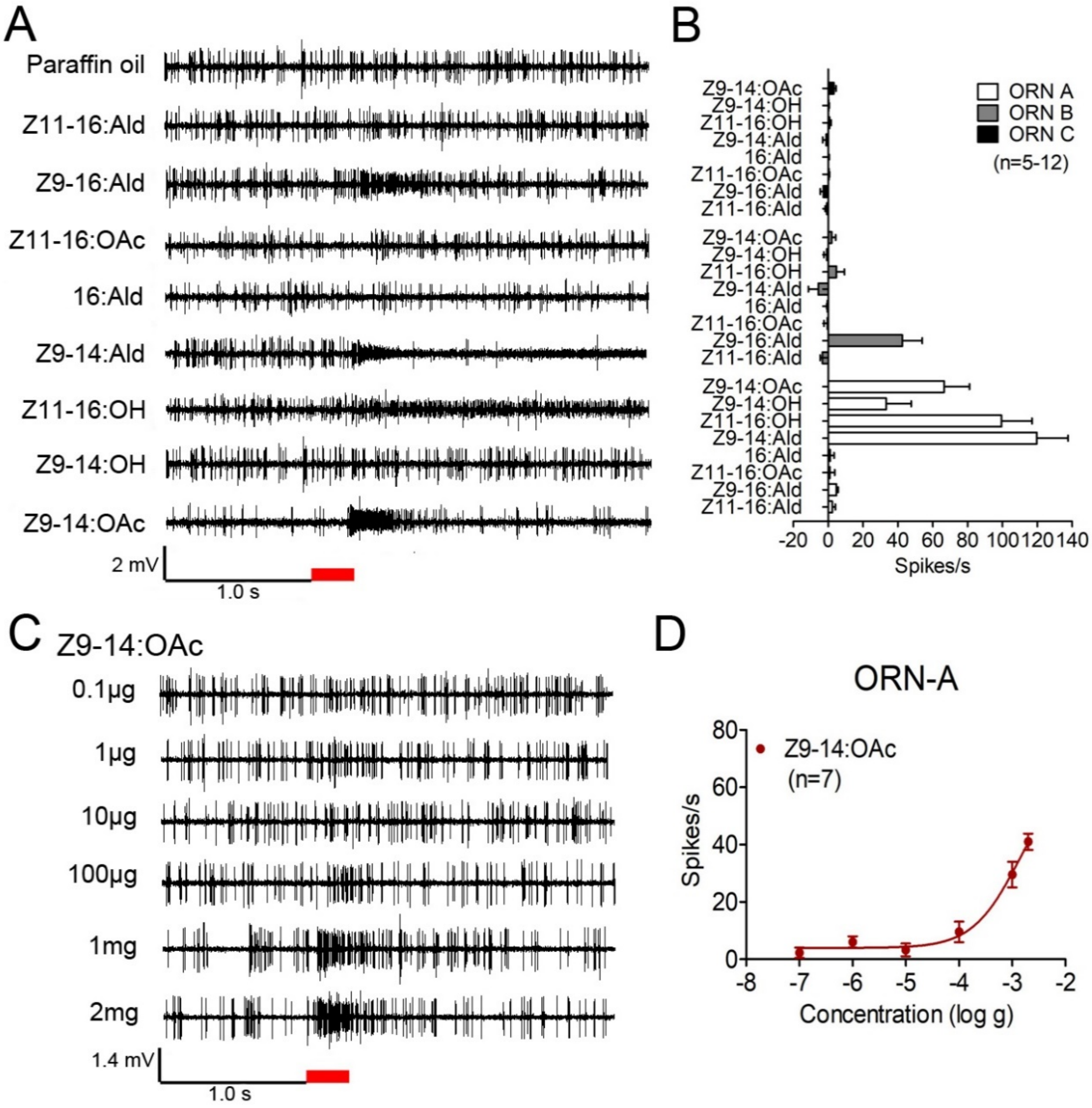

Figure S2. Response profiles of type-II AT in sub-group 2 activated by Z9-14:OAc on the antennae of male *M. separata*. (A) SSR traces showing ORN-A were activated by Z9-14:Ald, Z11-16:OH, Z9-14:OAc and Z9-14:OH. ORN-B were activated by Z9-16:Ald. (B) Response profiles of distinct ORNs housed in these AT. ORN-A was activated by Z9-14:Ald, Z11-16:OH, Z9-14:OAc and Z9-14:OH with responses of 120 ± 18 spikes/s, 100 ± 18 spikes/s, 66.50±14.60 spikes/s and 33 ± 14 spikes/s, respectively. ORN-B was activated by Z9-16:Ald with responses of 43 ± 12 spikes/s. Error bars indicate SEM (n=5-12). (C) SSR traces showing ORN-A were activated by Z9-14:OAc across a range of doses from 0.1 μg to 2 mg. (D) Dose-response curves of ORN-B. Error bars indicate SEM (n=7).

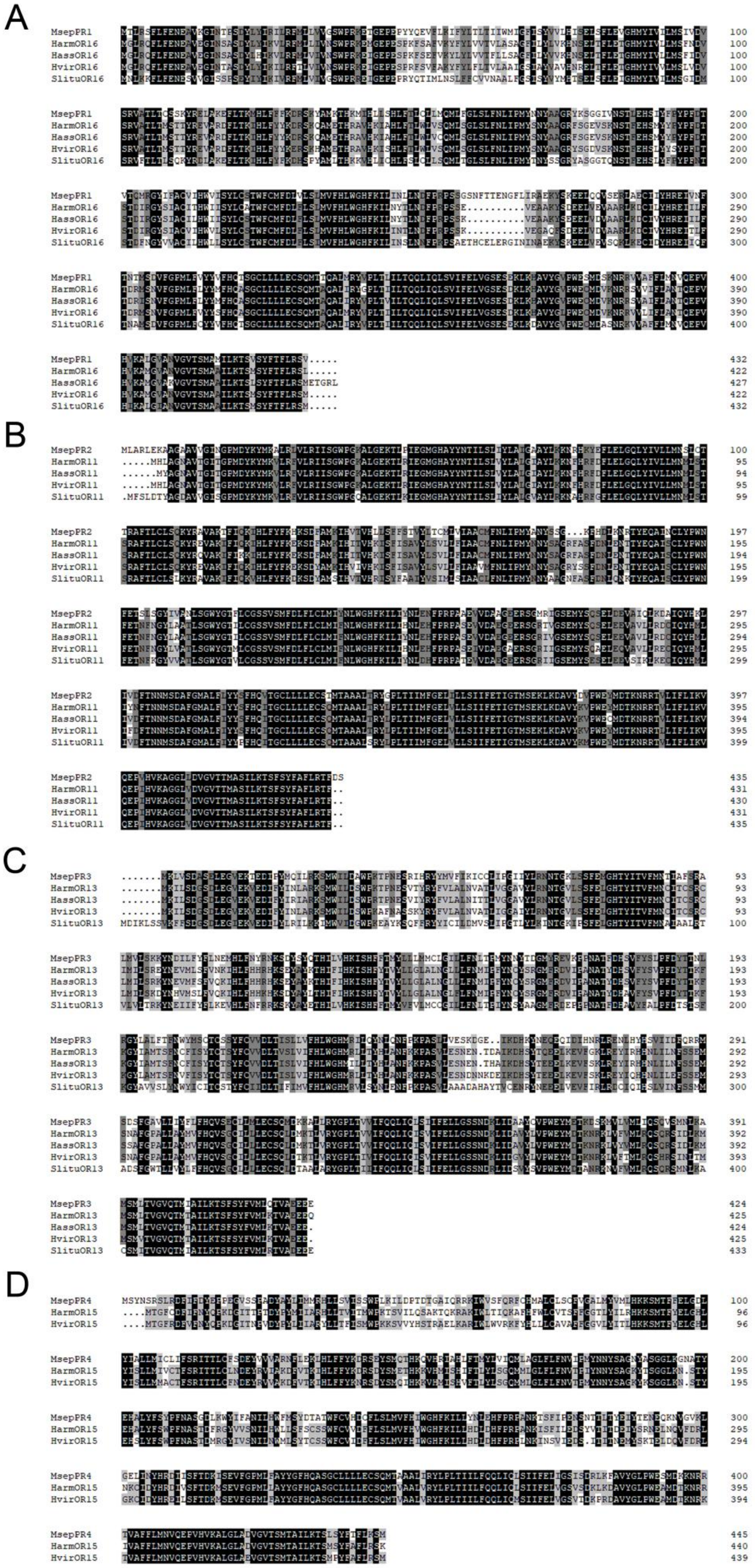
A
MsepPR1
HarmOR16
HassOR16
HvirOR16
SlituOR16
B
MsepPR2
HarmOR11
HassOR11
HvirOR11
SlituOR11
C
MsepPR3
HarmOR13
HassOR13
HvirOR13
SlituOR13
D
MsepPR4
HarmOR15
HvirOR15

Figure S3. (A) Amino acid sequence alignment of MsepPR1 with homologous OR16. Identities of identical amino acids range between 70.02% and 73.61%. (B) Amino acid sequence alignment of MsepPR2 with homologous OR11. Identities of identical amino acids range between 80.59% and 81.96%. (C) Amino acid sequence alignment of MsepPR3 with homologous OR13. Identities of identical amino acids range between 61.43% and 63.51%. (D) Amino acid sequence alignment of MsepPR4 with homologous OR15. Identities of identical amino acids range between 66.07% and 67.42%.

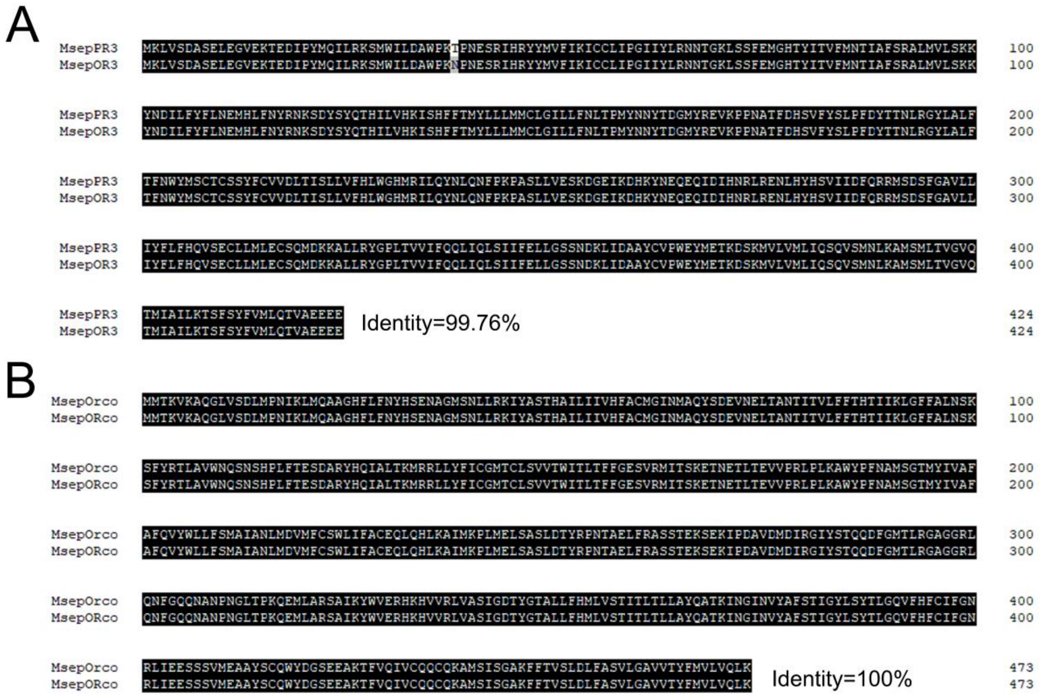

Figure S4. (A) Amino acid sequence alignment of MsepPR3 (Xinxiang geographic population) with MsepOR3 (Baoding geographic population). (B) Amino acid sequence alignment of MsepOrco (Xinxiang) with MsepORco (Baoding).

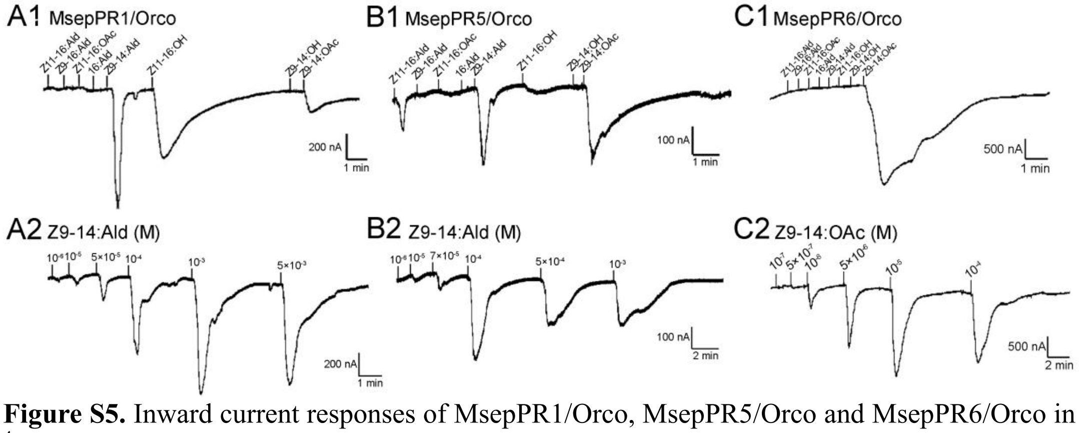

Figure S5. Inward current responses of MsepPR1/Orco, MsepPR5/Orco and MsepPR6/Orco in response to 10^{-4} M solution of four sex pheromone components and four pheromone analogues (A1, B1, C1). Dose-responses of MsepPR1/Orco, MsepPR5/Orco and MsepPR6/Orco expressed in *Xenopus* oocytes stimulated with a range of Z9-14:Ald, Z9-14:Ald and Z9-14:OAc concentrations, respectively (A2, B2, C2).

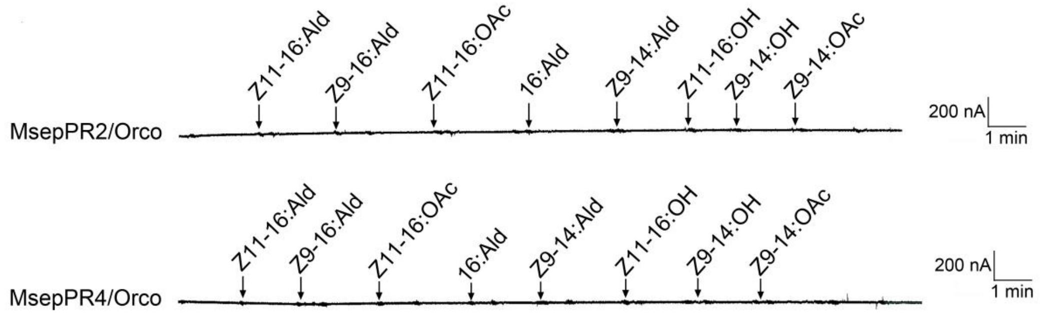

Figure S6. Response profiles of MsepPR2/Orco and MsepPR4/Orco to sex pheromone components and their analogues.
